# Light-driven soft microrobots based on hydrogels and LCEs: development and prospects

**DOI:** 10.1039/d4ra00495g

**Published:** 2024-04-29

**Authors:** Yingnan Gao, Xiaowen Wang, Yibao Chen

**Affiliations:** a School of Electromechanical and Automotive Engineering, Yantai University Yantai 264005 China 17865570339@163.com WXW2435461356@163.com ytuchenyb@163.com

## Abstract

In the daily life of mankind, microrobots can respond to stimulations received and perform different functions, which can be used to complete repetitive or dangerous tasks. Magnetic driving works well in robots that are tens or hundreds of microns in size, but there are big challenges in driving microrobots that are just a few microns in size. Therefore, it is impossible to guarantee the precise drive of microrobots to perform tasks. Acoustic driven micro-nano robot can achieve non-invasive and on-demand movement, and the drive has good biological compatibility, but the drive mode has low resolution and requires expensive experimental equipment. Light-driven robots move by converting light energy into other forms of energy. Light is a renewable, powerful energy source that can be used to transmit energy. Due to the gradual maturity of beam modulation and optical microscope technology, the application of light-driven microrobots has gradually become widespread. Light as a kind of electromagnetic wave, we can change the energy of light by controlling the wavelength and intensity of light. Therefore, the light-driven robot has the advantages of programmable, wireless, high resolution and accurate spatio-temporal control. According to the types of robots, light-driven robots are subdivided into three categories, namely light-driven soft microrobots, photochemical microrobots and 3D printed hard polymer microrobots. In this paper, the driving materials, driving mechanisms and application scenarios of light-driven soft microrobots are reviewed, and their advantages and limitations are discussed. Finally, we prospected the field, pointed out the challenges faced by light-driven soft micro robots and proposed corresponding solutions.

## Abstract 2

In this paper, the driving materials, driving mechanisms and application scenarios of light-driven soft microrobots are reviewed, and their advantages and limitations are discussed. Finally, we pointed out the challenges faced by light-driven soft micro robots and proposed corresponding solutions.

## Graphical abstract

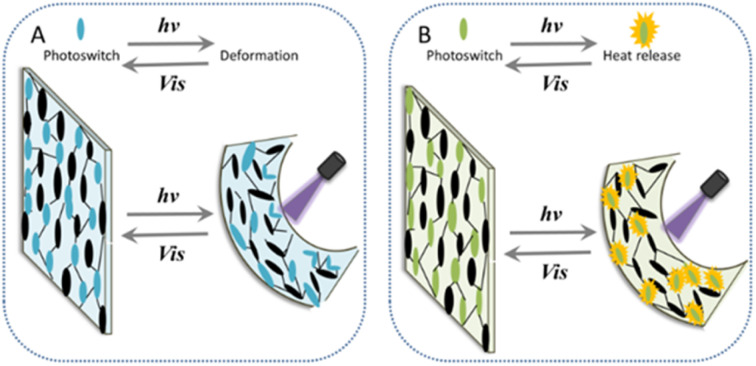

## 1. Introduction

Microrobots are robots that range in size from centimeters to nanometers. With the development of robotics and micro technology, scientists from all over the world have gradually paid attention to the research of microrobots and constantly promoted the development of them.^1^ The current research in this field has made great progress and successfully demonstrated its wide application.^2^ Light can be used to transmit energy and information, so it is very versatile and can be used in integrated circuits, material processing, single molecule detection and laser techniques. Light drive is one of the most common methods for controlling mobile microrobots.

At present, researchers have made many microrobots by using photocatalytic and photomechanical materials, and the movement of microrobot can be controlled by illumination accurately.^3^ Light-driven soft microrobots are those rely on the interaction between photosensitive materials, such as liquid crystal polymers and hydrogels.^4^ The micromanipulator based on this principle is widely used for picking and placing objects. In addition, it can also be used to make flowers, Venus flytraps and other bionic soft robots.

Section 2 introduces the fabrication materials for light-driven soft microrobots;^5^ in Section 3, two kinds of driving mechanisms of light-driven soft microrobot are introduced, that is, photothermal drive and photomechanical drive, and Section 4 describes the related applications of light-driven soft microrobots, including bionics, intelligent transportation, environmental protection, and surface cleaning.^6^

Finally, we summarize the challenges faced by light-driven microrobots and look forward to their development prospects. It is hoped that the driving mechanism described in this paper will arouse readers' interest and stimulate readers to further explore the manufacturing materials of light-driven microrobots and the functional application of related materials.^7^

## 2. Materials and methods

When the concept of soft microrobots was first proposed, the commonly used materials were mainly traditional hard materials, such as metal, plastic and silicone.^8^ Although these materials have certain toughness and plasticity, it is difficult to achieve true flexibility and bending deformation.^9^ With the development of materials science and nanotechnology, a new generation of flexible materials and flexible electronic materials have been applied to the manufacture of soft microrobots. These materials have a high degree of flexibility, scalability, electrical conductivity and biocompatibility, meeting the new demand for material properties in soft microrobots. The most commonly used materials for manufacturing light-driven soft microrobots are hydrogels and liquid crystal elastomers (LCEs). Hydrogels and LCEs have excellent flexibility and deformation characteristics, which can be used to manufacture flexible sensors of soft microrobots, realizing sensitive detection and measurement the pressure, deformation and other parameters. There is a broad development space for the design and application of soft microrobots.

### 2.1 Hydrogel

Hydrogel is a kind of gel with water as dispersing medium, which is a kind of polymer three-dimensional network system.^10^ It is soft and deformable with good water absorption and can maintain a certain shape.^11^ Light-driven soft microrobots based on hydrogels have many advantages, such as ease of synthesis and the ability to adjust the low critical solution temperature (LCST, is about 32 °C) by modifying the hydrophilic and hydrophobic balance within the side chain groups. Conventional hydrogels are not sensitive to environmental changes such as temperature or pH. However, the new hydrogel has environmental sensitivity, and can sense small changes or stimuli in the external environment (such as temperature, light, pH, humidity, pressure, *etc.*), and can produce corresponding changes in physical structure and chemical properties. Therefore, the new hydrogel has good environmental responsiveness, which can be used as sensors, controlled release switches, microrobots, *etc.*.

Poly(*n*-isopropylacrylamide) (PNIPAM) is a temperature-responsive hydrogel, which has both hydrophilic acyl groups and hydrophobic isopropyl groups on the macromolecular chain.^12^ It is notable for its ability to undergo a phase transition when the temperature changes. During the polymerization process, swelling ratio of PNIPAM can be controlled by controlling parameters such as monomer ratio, oxygen concentration and initiator concentration.^13^ In practical applications, PNIPAM can be used to prepare carriers such as microspheres and nanoparticles for targeted drug delivery. By controlling the temperature to control the release rate of the drug, thus realizing the drug delivery at a fixed point in a quantitative manner. In addition, PNIPAM can also be used to prepare biological scaffolds and artificial organs, *etc.*, alleviating the current clinical problem of organ shortage. Temperature–responsive properties allow PNIPAM to be used in many applications such as targeted drug delivery, tissue reconstruction, *in vivo* imaging, and temperature-controlled valves, *etc.* PNIPAM are mainly made by polymerizing photo-initiators, photo-cross-linkers, and *N*-isopropylacrylamide (NIPAM) monomers.^13^

Pan *et al.* prepared stimuli-responsive bilayer actuators based on PNIPAM hydrogels.^13^ The structure was made stimuli-responsive by doping the polymeric initiator and stimuli-responsive nanomaterials directly into the bilayer. The structure can deform with the change of humidity and illumination. Pan *et al.* developed a bionic soft microrobot with grasping, crawling and jumping functions based on this actuator, which is applied to environmental monitoring or drug delivery in biomedical engineering.

Rehor *et al.* developed micrometer-sized hydrogel crawlers based on PNIPAM hydrogels, which achieve movement in liquid environments by friction between their bodies and the substrate. The loaded gold nanoparticles endowed the hydrogel crawlers with photoresponsive properties, allowing them to contract reversibly under localized laser irradiation. The non-uniform deformation of the hydrogel leads to asymmetric friction between the crawler and the substrate, which results in directional movement of the robot. The robot can act as a micromanipulator arm to propel small cargoes along the surface.^10^

Hydrogels have excellent flexibility and can be adapted to a wide range of shapes and environments, allowing the fabrication of soft microrobots that can move and perform tasks in complex environments. Some kinds of hydrogels are also biocompatible and can be used in biomedical applications such as medical diagnostics and drug delivery.^14^ The mechanical properties of hydrogels can also be controlled by adjusting their composition and structure. However, some hydrogels have poor durability and lack mechanical strength, easily affected by environmental factors. And hydrogels cannot work without water.^15–21^ In recent years, researchers have also explored hydrogels with shape memory, but most of them have to be used in a bilayer or multilayer structure, which cannot avoid the phenomenon of delamination. Moreover, the volume change of hydrogel is isotropic, limiting the movement modes of hydrogel microrobots. Because of the above reasons, researchers have turned their attention to LCEs with anisotropic properties, which can deform in the absence of water.

### 2.2 LCEs

The other kind of material often used to fabricate light-driven soft microrobots is LCEs.^22^ The researches of liquid crystals originated in the 19th century, and it was noticed that LCEs were intermediate between solid crystalline and liquid, with molecular arrangements which were neither ordered nor disordered as in the solid crystalline state. These polymers are ordered and stimulus responsive.

The first liquid crystal display was introduced in the 1970s. It greatly promoted the in-depth study of the properties and molecular structure of liquid crystal materials. LCEs refer to liquid crystal polymers which display elasticity in the isotropic state or liquid crystal state, and have the dual characteristics of liquid crystal and elastomer after moderate cross-linking. It not only retains the properties of the original non-crosslinked liquid crystal polymers, but also has great potential in the fabrication of soft microrobots, wearable devices, artificial muscles and bionic devices due to the excellent orientation, piezoelectricity, ferroelectricity, soft elasticity under the effect of mechanical force field, as well as the huge reversible driving force generated by deforming.^23,24^

Although LCE loses the rigidity of solid substances, it gains the fluidity of liquids, and retains the anisotropic ordered arrangement of some crystalline substance molecules, so it makes an intermediate state with some properties of crystals and liquids. Under external stimulation, LCE can change from order to disorder. Based on this property, researchers have created a variety of stimulus-responsive LCE microrobots.^25^ Pang *et al.* made voltage-driven soft actuators based on LCEs that can realize different motion gaits.^26^ By combining deformable foot pads and smart joints, Pang *et al.* reported a multi-gait microrobot capable of crawling on planar, cylindrical, wavy, wedge-shaped groove and spherical surfaces, respectively. The soft microrobot is able to climb over obstacles, operate in confined spaces, and also climb along vertical directions. Subsequently, Shi *et al.* created a self-oscillating microrobot powered by a broad spectrum of constant light, which consists of a LCE layer containing candle soot (CS) and a polydimethylsiloxane (PDMS) layer.^27^ A bar oscillator fixed at one end can perform self-sustained oscillations by the self-shadowing mechanism. The LCE layer containing CS as an excellent light absorber provides excellent photothermal drive ability. The PDMS layer with low viscoelasticity accelerates the drive-recovery cycle of the oscillator. The LCE composite optical oscillator displays tunable frequency and amplitude through structural and optical intensity modulation, showing potential for autonomous soft robotics applications. In addition, HE *et al.* has successfully built micro-tweezers, microrobots and photo-powered microfluidic pumps using electrospinning techniques.^28^

**Fig. 1:**
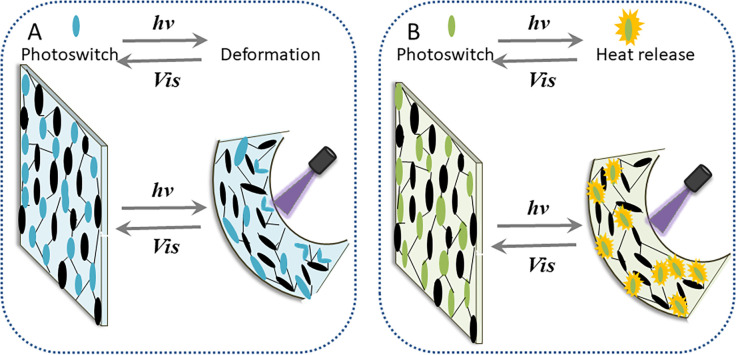
(A) Opto-mechanical drive. (B) Photothermal drive.

Researchers endow LCEs with stimulus–responsive properties by introducing photo-switches. Wang *et al.* categorized optical switches into photo-thermal and photo-mechanical optical switches based on their operating principles, and then categorized LCEs robots into photo-thermal and photo-mechanical driving mechanisms.^29^ In photo-thermal driving mechanism, photo-thermal switches endowed into LCEs are carbon materials, metal nanomaterials, *etc.*. These photo-thermal switches convert light energy into heat energy, which dissipating into the surrounding LCE network, causing the molecular sequence in it to change from ordered to disordered, leading to the shape change of the LCE, as shown in Fig. 1A. Photo-mechanical driving mechanism is to dope photo-mechanical switches such as azobenzene in the LCEs, which can absorb light energy and change its own volume, stressing the surrounding molecular network of LCE and causing macroscopic volume changes of LCEs (Fig. 1B). In addition, a few photo-mechanical switches operate similarly to photo-thermal switches, so that the generation of heat and stress cause the deformation of LCEs together.^30^ In Section 2.1, the microrobots based on hydrogels we mentioned can basically only be driven in a liquid environment or at the gas–liquid interface, so the working environment is seriously limited. Compared with hydrogels, microrobots made based on LCEs can be driven in complex media or air, so the application range is much wider. In addition, LCEs are more elastic and plastic, so the lifespan of LCEs microrobots is longer.^31^

## 3. Driving mechanisms

### 3.1 Driven by the photothermal effect

#### 3.1.1 Hydrogel soft microrobot driven by photothermal effect

As an external stimulus or energy source, light can drive the movement of microrobots with the advantages of dynamic programming, wireless, on-demand remote control, high spatial and temporal resolution.^32^ The response principle of photo-responsive hydrogels to light is divided into two types, the first is to add photo-thermal conversion materials (such as nanoparticles, carbon nanoparticles, *etc.*) to the hydrogel. When composite material is exposed to light, the hydrogel does not respond to light, but the photo-thermal conversion material in it absorbs and converts light energy into heat energy, causing the internal network of the illuminated side to shrink, and the water in this side lost. While the volume of the backlight side remains unchanged, causing the hydrogel to bend toward the illuminated side, and thus triggering a series of movements. Second, light causes the break and recombination of macromolecular chains inside the hydrogel, thus changing the cross-linking density of hydrogen,^33^ which leads to local volume changes of the hydrogel, resulting in the occurrence of non-uniform deformation and triggering movement. The first method is the main driving principle of light-driven soft microrobot because of its simple principle, simple process flow, low cost and wide applicability. Therefore, in this part, we mainly introduce hydrogel soft microrobots driven based on first principles.

**Fig. 2:**
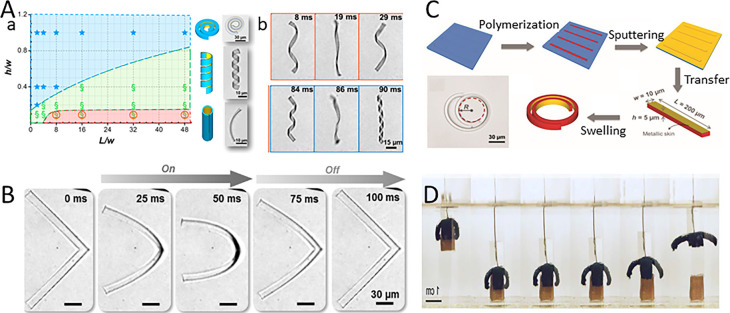
(A) The shape of the double-layer hydrogel bands varies with different sections and lengths, and the unwinding and reversal of the helix can be completed within 90 milliseconds (this figure has been reproduced from ref. 34 with permission from ACS Publications, copyright 2024). (B) Various states of L-type microgels in a modulation cycle (this figure has been reproduced from ref. 35 with permission from Wiley-VCH, copyright 2024). (C) Preparation of bilayer spiral microhydrogels (this figure has been reproduced from ref. 37 with permission from John Wiley and Sons, copyright 2024). (D) Grasping objects using soft micro-robots made with a PVA passivation layer (this figure has been reproduced from ref. 36 with permission from Science Robotics, copyright 2024).

In a typical example, Zhang *et al.* reported a PNIPAM strip coated with a thin gold layer.^34^ Illuminating infrared light on the gold layer, light energy converts into heat energy, causing the hydrogel bands to curl. The results show that the shape of bilayer strip with different cross sections and lengths will have different degrees of crimp as shown in Fig. 2A-(a). Zhang *et al.* also found that the unwinding and reversal of the spiral can be accomplished within 90 milliseconds as shown in Fig. 2A-(b).^34^ The experimental results show that the dynamic deformation of hydrogels can be applied to microrobots, microfluidics and artificial microfluidics.

Mourran *et al.* designed a light-controlled microswim device that can transform its body to drive rotation and progress, by laser heating of gold nanorods embedded in PNIPAM hydrogel, the volume of the thin hydrogel is rapidly expanded, thereby driving the bending and twisting of the microrobot. When the laser is removed, the microrobot returns to its original structure (Fig. 2B).^35^ In addition, Hippler *et al.* describe a 3D heterogeneous photostimulus response valve based on PNIPAM and *N*-methylene bisacrylamide (*N*,*N*′-methylene bisacrylamide).

As shown in Fig. 2C, Zhang *et al.* designed a micron-sized bilayer banded robot by using PNIPAM hydrogel.^35^ One layer of the bilayer structure is loaded with gold nanorods, which are used to convert near-infrared light energy into local heat, triggering the change of volume. The other layer contains no gold carbon nanorods, whose volume does not change when exposed to light. The volume difference between the two layers causes the bending deformation of the bilayer structure. A silicon master mold made according to photolithography replicates the non-wetting soft mold. Isolated microgel particles containing AuNRs can then be molded into the desired shape within the soft template by photopolymerization. After polymerization, the top surface of the microgel is coated with a gold skin, and the microgel is then released into water through a transfer process. When swelling in water, the expansion of the gel layer is limited by the gold skin, which prevents stretching. As a result, the swelling of the hydrogel produces different strains, leading to bending of the bilayer. Depending on the initial geometry of the ribbon material, different structures can be formed, such as helixes or two-dimensional spirals. This work sheds new light on artificial micro-swimmers, and the design principle can also be extended to program other moving elements for biomedical and microfluidic applications, as well as for the development of new soft microrobots.

Zhao *et al.* proposed that simultaneous actuation and sensing can be achieved by combining a stimulus-responsive hydrogel with conductive polymer molecules in a single material as a model system as shown in Fig. 2D.^36^ This homogeneous bulk conductive hydrogel consists of an interpenetrating polymer bi-network of PNIPAM and polyaniline (PAni), which combines photo-thermo actuation and piezoresistive sensing into a monolithic material with a “two-in-one” functionality. By assembling four hydrogel arms, Zhao *et al.* succeeded in creating a cross-shaped soft fixture that can quickly grasp objects in hot water. A continuous snapshot of a gel with a polyvinyl alcohol (PVA) passivation layer grasping an object in hot water at 45 °C is shown. This directed asymmetric motion is attributed to the temperature gradient on the hydrogel, where the temperature on the illuminated side increases above the LCST while the temperature on the shadowed side remains below the LCST, leading to localized contraction on the illuminated side and overall bending to grasp the object.

Local contraction of the hydrogel results in asymmetrical changes in friction between the tractor and the substrate. The track generates directional motion through eccentric irradiation, making the track push small loads on a flat surface like a micromanipulator. Responsive hydrogel actuators have good application prospects in various fields. However, typical hydrogel drives usually need to be implemented in water, and deformation drives cannot be implemented in air or dry environments.

#### 3.1.2 LCEs soft microrobot driven by photothermal effect

Soft microrobot driven by photo-thermal effect utilizes the absorption of light energy of liquid crystal material to generate heat, driving the motion of microrobot.^38^ This kind of robot uses light energy as an energy source. By controlling the position and intensity of light illumination point, the movement and operation of the microrobot realize. This kind of robot is characterized by small size, high flexibility, low energy consumption, *etc.*, and has a wide range of application prospects in the micro and nano fields. Continuously explore the development of more efficient liquid crystal materials to improve the efficiency of photo-thermal conversion, reduce energy loss, as well as improve the toughness and stability of the material. In recent years, the liquid crystal soft microrobot based on photo-thermal drive is gradually developing in the direction of intelligence, integration and mass production.^39^

Compared to hydrogel robots, the material properties of liquid crystals enable the robots to exhibit better deformation and motion characteristics under photothermal stimulation, realizing flexible morphology transformation and traversal of tiny spaces. The photothermal effect can provide the driving force at a tiny scale, enabling the robot to realize micromanipulation and localization.^40^

**Fig. 3:**
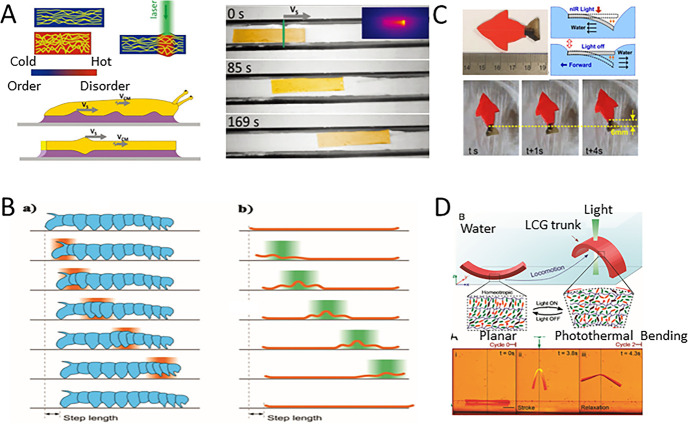
(A) The crawling of bionic snails (this figure has been reproduced from ref. 40 with permission from Macromolecular Rapid communications, copyright 2024). (B) The crawling of bionic caterpillars (this figure has been reproduced from ref. 42 with permission from American Chemical Society, copyright 2024). (C) Swimming of a bionic fish (this figure has been reproduced from ref. 43 with permission from American Chemical Society, copyright 2024). (D) The Red Sea bass swims with the curvature and undation of its central torso (this figure has been reproduced from ref. 46 with permission from PANS, copyright 2024).

Rogoz *et al.* combined spatially and temporally modulated laser beam-induced deformation of a soft elastomer with the adhesion properties of a synthetic mucus.^41^ A light-driven millimeter-scale crawling robot was demonstrated, as shown in Fig. 3A, mimicing the locomotion patterns of terrestrial gastropods such as snails. They utilized the photo-thermal sensing of a LCEs as an actuator, similar to a crawler robot. The robot body contracts along its long axis during movement while remaining flat, and the body is always in contact with the entire moving surface through a mucus layer. The designed bionic robot can move smoothly on a variety of surfaces and can even move upside down on glass or ceilings.

In addition, Rogoz *et al.* also designed a soft microrobot based on liquid-crystal elastomers based on the motion gait of a caterpillar, as shown in Fig. 3B.^42^ The caterpillar has a soft cylindrical body with three pairs of thoracic legs on the front, two to five pairs of soft legs on the abdomen, and terminal legs on the back. In each crawl cycle, the terminal legs are first released from the ground and moved forward to re-engage with the substrate. Each leg on the abdomen is lifted in turn, taking a step forward to form a wave-like pattern from the tail to the head. Rogoz *et al.*'s use a spatially modulated light field to trigger deformations in different parts of the robot's body. The laser beam is first projected onto the tail of the caterpillar (the trailing end of the stripes), which absorbs the light and morphs into a curved shape while rising up from the ground and shortening. The laser beam then scans from the tail end toward the robot's head, causing the caterpillar to move and deform accordingly, just like a crawling caterpillar. After the beam completes its scan, the robot returns to its original flat state, one step forward completes.

Tian *et al.* fabricated a soft fish-shaped swimming robot with a thickness of 1 mm,^43^ whose body and caudal fin were made of a polyacrylate (VHB) membrane that simply adhered to a polydopamine (PDA)-coated LCE membrane. Due to the photothermal effect of the PDA coating and the thermal responsiveness of the LCE, the elastomer membrane shrinks significantly under the illumination of near-infrared (NIR).^44^ The swimming motion was powered by the momentum exchange between the solid tail fin and the ambient fluid. As the Fig. 3C shown, when the robot is exposed to NIR light, the surface LCE membrane heats up, causing the tail to bend downward. After turning off the NIR light, the restored elasticity of the robot's tail rapidly drives the tail upward and squeezes the fluid backward, effectively generating thrust that propels the robot forward.^45^

In addition, Shahsavan *et al.* were inspired by sea slugs and snails and used liquid crystalline gels (LCG) to create artificial structures with aquatic motility mechanisms. Fig. 3D shows a schematic of an artificial monomer with a mixed molecular arrangement that mimics the undulation and curvature of a sea slug's torso in a simple manner.^46^ In this model, photothermal induction causes the molecules to change from order to disorder to store elastic energy, and the torso recovers the relaxed energy after stopping the light, so that the motion can be achieved through repeated compression and relaxation of the local curvature. Left side corresponds to an open molecular arrangement, while the right side corresponds to a photothermally deformed LCG structure with disordered molecules. Fig. 3D-(b) shows LCG's cantilever structure achieving continuous upward motion in the water in a continuous lighting cycle.

### 3.2 Driven by photomechanical effects

Photomechanical switches can change their shape by absorbing light, and photomechanical switches (such as azobenzene, *etc.*) can be added to the liquid crystal to make microrobots.^47^ When illuminated, these photo-mechanical switches exert stress on the surrounding LCE molecular network, causing the molecules to change their original arrangement shape and change their macroscopic volume.

**Fig. 4:**
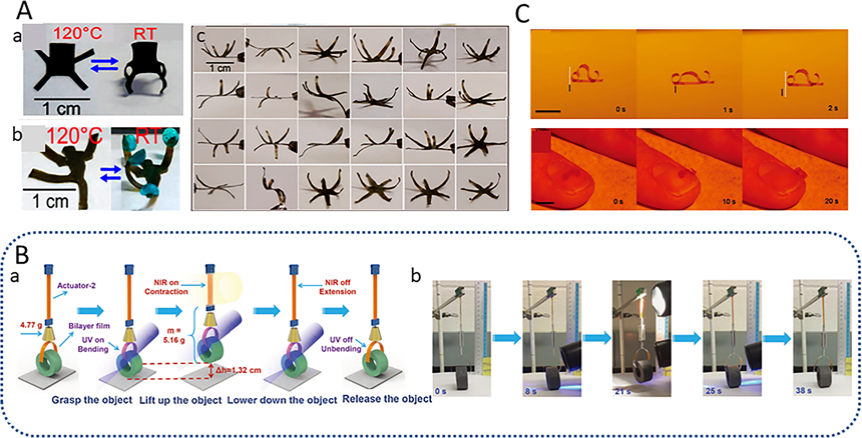
(A) (a) Reversible changes in the shape of liquid crystal polymer chairs. (b) Liquid crystal Hercules can lift up to four balls under light (this figure has been reproduced from ref. 48 with permission from American Chemical Society, copyright 2024). (B) The polymer “crane” performs a series of motion tasks of a combined light-driven robot, including grasping, lifting, lowering, and releasing a tubular object (this figure has been reproduced from ref. 50 with permission from Advanced Materials, copyright 2024). (C) Light-driven movements on paper surfaces and human fingernails (this figure has been reproduced from ref. 49 with permission from Macromolecular Rapid Communications, copyright 2024).

It is difficult to achieve 3D deformation in dry environments. Yang *et al.* created an LCE-based chair, which is a two-dimensional structure when there is no illumination, and when locally illuminating the LCE film containing carbon nanotubes (CNTs) with infrared light caused the four chair legs to bend downward and the back of the chair to bend upward, whereupon the film was formed into a chair as shown in Fig. 4A-(a). They also used the same principle to create a strong man who can lift heavy objects many times larger than himself as shown in Fig. 4A-(b). They also used the same principle to create strong men capable of lifting heavy objects many times heavier than themselves as shown in Fig. 4A-(b). CNT-xLCE dynamic 3D structures can be easily remodeled, reconfigured, and even recycled.^48^ Yang *et al.* also designed a programmable flower as shown in Fig. 4A-(c), where a six-petaled “flower” is created by aligning each of the six petals. As shown in Fig. 4A-(c), a six-petal bionic flower is made into more than 20 different shapes by aligning each petal to contract or bend in different directions.

**Fig. 5:**
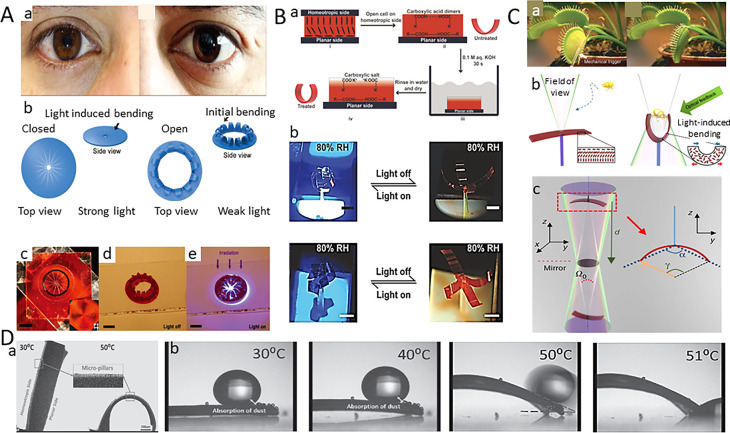
(A) Iris production process. This iris opens in the dark and closes in light (this figure has been reproduced from ref. 55 with permission from Advanced Materials, copyright 2024). (B) (a) The LCE band is soaked in an alkaline solution to make it sensitive to humidity. (b) The treated LCE strip has less curvature at low humidity (this figure has been reproduced from ref. 56 with permission from Advanced Materials, copyright 2024). (C) (a and b) a picture of the fly trap and the principles for capturing the object. (c) Leaves a hole in the center of the LCEs through which light is emitted (this figure has been reproduced from ref. 59 with permission from nature communications, copyright 2024). (D) Schematic diagram of heat-induced deformation of LCE films. When the heat-induced curvature reaches the slip Angle, the glycerol droplets slide off the film along the shortest path (this figure has been reproduced from ref. 58 with permission from Europe PMC, copyright 2024).

Applying photochemical reactions and photothermal effects to control optomechanical actuation in a device to perform complex motion tasks and perform useful work remains a major scientific challenge. Lu *et al.* demonstrated a solution to the challenge by doping polymer-grafted gold nanorods into dynamic azobenzene liquid crystal networks, which endowed the polymer with near-infrared and ultraviolet photo-responsive properties, thus enhancing the photocontrol of actuation as shown in Fig. 4B. Finally, Lu *et al.* combined the effects of two light-triggered molecular changes, *i.e.*, the isotropic phase transition of liquid crystals and the photoisomerization of azobenzene. Based on this, a polymer “crane” was designed that is capable of complex, synergistic, robot-like macroscopic motions controlled by light. This work not only designs a new fabrication method for light-responsive LCE actuators based on two mechanisms, but also takes an important step forward in the potential application of artificial muscles and bionic soft robots as shown in Fig. 5B.^49^

## 4. Applications

### 4.1 Bionic technology

Biology itself has much superior functions than any artificial robot, and bionic technology is to achieve these functions in engineering, and it is reasonable to apply it to life practice. The term bionics was coined by American scientist Steele in 1960 based on the Latin word “bios” and the “nic” (having… is a combination of…).^51^ The creature has features that are far superior to robot, such as a unique outline or movement form. But robots also have advantages that creature can't match, such as repeatability and accuracy. Therefore, more and more researchers combine bionic technology with robot technology to make bionic microrobot.^52,53^ These bionic robots combine the advantages of both biological and machine, and have significant applications in human production and life. Bionic technology is based on the research of organisms, providing new design inspiration and working principle for engineering.^54^ The study of bionic technology opens up a new way of technological development and greatly broadens people's vision. In recent years, with the development of light stimulation technology and micro-fabrication technology, many researchers have integrated the both to fabricate light-driven microrobots.^14^

The iris, which is present in many animal species. It can control the light intensity entering the eye by changing the size of the pupil to cope with different lighting conditions. Fig. 5A-(a) LCEs bionic iris designed by Zeng *et al.*, whose shape can be automatically adjusted according to the light intensity. The artificial iris has no control circuit, so it cannot zoom in and out on its own. Giving the artificial iris the ability to adjust its size autonomously according to light intensity is particularly important.^55^Fig. 5A-(b) shows the fabrication process of the bionic iris. First of all, two slides are placed together, the top is coated with an isotropic arrangement layer and the bottom is coated with a light arrangement layer, so that the substrate layer can be obtained. The LC monomer mixture is introduced into the substrate layer so that the composite obtains the ability to convert the linearly polarized beam into the azimuth-polarized beam. Then, the artificial iris is obtained by projecting laser beam onto the substrate coated with a light alignment layer to adapt the azobenzene molecules to the radial distribution. The artificial iris opens with the absence of light and closes in the presence of light. The experimental results show that the bionic iris has the same characteristics as the natural iris. Wani *et al.* utilized the light and wet dual response mechanism of LCEs to fabricate an artificial nocturnal flower. This bionic flower closes during the day with low humidity low or intense light, and open when humidity is high or light is low. Initially, the LCEs strip is soaked in an alkaline solution to make it sensitive to both light and humidity. The curvature of the treated LCE strips is small at low humidity and large at high humidity. Compared to conventional photo-thermal LCE actuators, the fuel of wet-gated photo-actuators is able to sense lower light intensity and can therefore be operated remotely through low light intensity as shown in Fig. 5B.^56^

Wani *et al.* designed and fabricated a light-driven flytrap the following year, which triggers a photo-mechanical actuation through optical feedback, as shown in Fig. 5C. The LCEs robot is attached to the tip of an optical fiber, whose functions like a probe for sensing the environment. An aperture is set in the center of robot, through which light supplied by optical fiber can be emitted. When an object enters the field of view and generates enough light feedback, the LCE bends and eventually captures the object. This artificial flytrap can perform the function of a natural flytrap, being able to recognize objects and close them autonomously. It is capable of self-driven regulation in a smaller frame, opening the way for soft and autonomous small devices (Table 1).

**Table 1:** Advantage and disadvantage of different driving mechanisms

Driving mechanism	Applicable microrobots	Working environment	Precision	Advantage	Disadvantage	Ref.
Photo-thermal driving mechanism	Hydrogel microrobot	Mainly liquids	High: 6 degrees of freedom	Convenient operation	Limited working environment	54 and 65–82
	LCEs microrobot	Air–liquid interface, air, liquid	High: 6 degrees of freedom	Convenient operation	Expensive to manufacture	24 and 83–86
Photo-mechanical driving mechanism	LCEs microrobot	Air–liquid interface, air, liquid	Low: limited by the properties of materials and design of microrobot	Large operational scalability	Expensive to manufacture; precision is limited by the properties of material and design of microrobot	22, 24, 58, 64, 83 and 87–93

### 4.2 Cleaning function

The powerful climbing abilities of geckos have been the subject of extensive study. Geckos can quickly stick their feet to smooth or rough surfaces, attribute to the switchable super-hydrophobic and self-cleaning properties of geckos' foot pads. From an anatomical viewpoint, the skin and muscles of the gecko foot pads have extremely effective adhesion and self-cleaning capabilities. At the skin level, nanoscale ciliated suckers on the gecko's toes promote intimate contact and maximize their interaction with the paired surface's van der Waals. The fibrous structure also has significant anti-fouling properties, which is so-called lotus leaf effect. Actually, the reverse roll of the toe not only disengages it from the contact surface, but also has enough acceleration to remove dirt articles. Researchers have created microrobots that can be used for surface cleaning by mimicking the climbing of geckos.^57^ Shahsavan *et al.* used the reversible shape changes of LCE to simulate the muscle movements of gecko toes. First of all, the LCEs layer is processed by integrated soft lithography to make the surface of it have microcolumns. The produced LCE sheet with a specific pattern can deform by heating.^55^

To verify the self-cleaning ability of LCE film, Shahsavan *et al.* placed small drops of glycerin on it. The film deforms as the rise of temperature, and when the bending curvature reaches a certain Angle, the particles on the film slide down the film along the shortest path with the glycerol drops. The stimulus–response properties of LCEs show great potential for further utilization own to their dynamic self-cleaning properties, as shown in Fig. 5D.^58^

### 4.3 Intelligent transportation

**Fig. 6:**
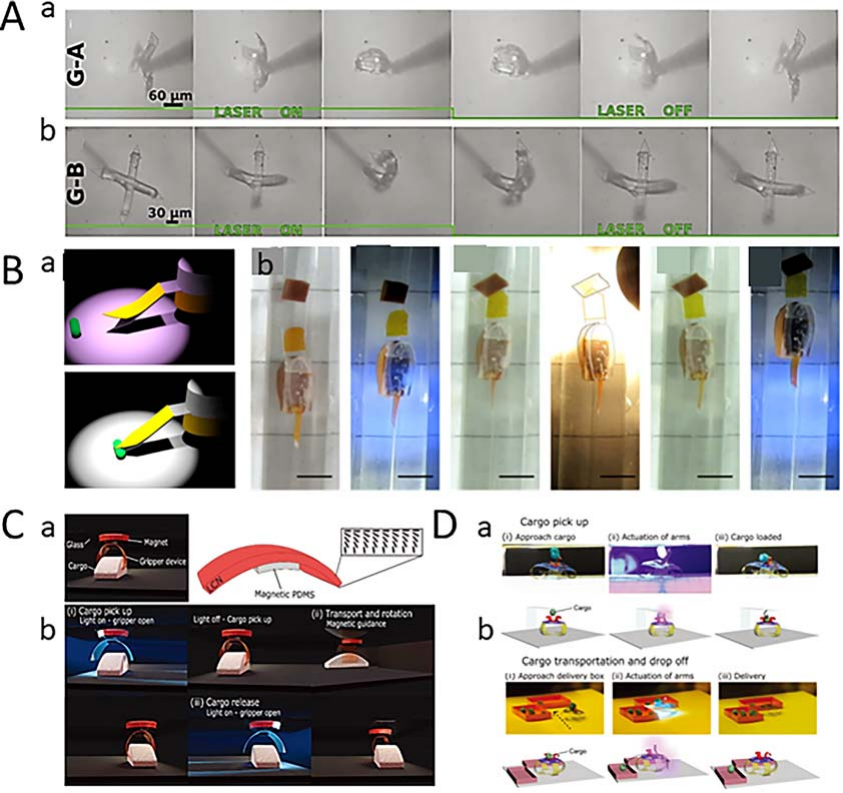
(A) The shape change of two microhands under laser irradiation (this figure has been reproduced from ref. 62 with permission from Advanced Materials, copyright 2024). (B) Robots use grippers to grab, transport and transport goods (this figure has been reproduced from ref. 63 with permission from Advanced Optical Materials, copyright 2024). (C) The light-driven gripper holds the object to realize intelligent transportation (this figure has been reproduced from ref. 63 with permission from Advanced Optical Materials, copyright 2024). (D) The four-claw microgripper moves to the target position and realizes the pick-up and placement of the object under the drive of the light (this figure has been reproduced from ref. 64 with permission from Advanced Science, copyright 2024).

Grasping and transporting tiny objects is a complex but indispensable function of microrobot. Inspired by the human hand and arm, a microscopic bionic hand capable of capturing tiny objects has been designed.^60^ The bionic hand is made up of light-sensitive soft materials, such as hydrogels and LCEs, which can be controlled remotely by light, automatically loading and unloading cargo based on the material's optical properties. The energy used to power the microrobot is transported optically, but without the use of wires or batteries.^61^ Compared with the traditional electric field controlled microrobot, this light-driven microrobot has the advantages of simple manufacture, excellent performance and easy control. Compared with the traditional electric field controlled microrobot, this light-driven microrobot has the advantages of simple manufacture, excellent performance and easy control. The bionic hand can shrink, which can be used for transportation in tiny environments, such as for transporting object in microfluidic devices. It is worth noting that the size of the “tiny space” here corresponds to the size of the microrobot. Microrobots can move in tiny spaces that cannot accommodate traditional robots, which may be centimeters-scale, or even micro-nano scale invisible to the naked eye. Of course, it also depends on whether the size of the microrobot is centimeter–millimeter or micro-nano scale. At present, researchers have simulated the movement of microrobots in artificial blood vessels with diameters of millimeters or even micrometers, and these microrobots must be millimeter or even micro-nano scale. In addition, there are also some centimeter scale microrobots that are used to work in narrow spaces at the centimeter scale. As early as 2010, Cheng *et al.* fabricated a light-driven microgripper based on CLCE/PE bilayer film for objects transporting. The microgripper consists of a CLCE layer and a polyethylene (PE) layer bonded together by an adhesive layer. Initially, the microgripper opens under vis. And then, the light is changed to illuminate the wrist, so that the hand is closed to the wrap object. After turning off the light, the microgripper grabs the object. Finally, the gripper opens again at the second light, and the object are also transport into the target container as shown in Fig. 6A. Huang *et al.* proposed a light-driven microrobot to load cargo in a liquid environment, which is made up of LCEs film sensitive to UV. Under UV illumination, the microgripper opens, and is driven to move towards the target objects by periodical light. When it reaches the target position, the microrobot is illuminated to close, realizing the capture of objects. Then the microrobot loading objects is transported to the target area by magnetic guidance, and the release of the objects is achieved by illumination as shown in Fig. 6B.^63^

Although the wireless microrobot described above can realize the grasp, capture, transportation and release of object, it can hardly be achieved by only one actuator. One year later, Cunha *et al.* reported a microrobot actuated by blue light, which consists of four LCE legs containing a yellow optical switch, two LCE arms containing a red optical switch and one LCE claw containing a red optical switch as shown in Fig. 6C.^63^ Compared with the LCEs leg containing the yellow optical switch, the LCEs arm containing the red optical switch is more absorbent to blue light and can fabricate greater deformation. By adjusting the position of the light illuminating on the LCE leg, the microrobot can walk by bending the legs alternately. When the LCE arm meets the suspended object, the object falls into the LCE claw. After removing the light, the LCE claw closes to wrap the object. When reaching the destination, the LCE claw opens by re-illumination. By illuminating light with low intensity on the LCE legs of the microrobot, its bent legs form a ramp to achieve object release. As shown in Fig. 6D, this study is a step forward in terms of actuator integration.^64^

## 5. Conclusions and challenges

Light is the most common energy source for wireless and remote drive micro-devices, which has been widely used as the driving energy for microrobots made up of photosensitive or photothermal materials.^94^ In this review, we introduce the working principle and material properties of light-driven soft microrobots. Then we demonstrate the wide applications of them in environmental protection, intelligent transportation, and bionic technology. It inspires us to explore the application of light-driven microrobots in multidisciplinary fields.

In the field of bionics, soft microrobots driven by light can imitate the structure and moving modes of natural organisms. Inspired by this, researchers have created artificial iris, artificial fly trap, artificial flower and so on. In addition, light-driven soft microrobot has inspired people in the aspects of information reception and information transmission. It provides new design ideas for engineering technology and is gradually applied to production practice.^95^

In the field of intelligent transportation, light-driven microrobots can efficiently capture and transport tiny objects to target area accurately. It has potential applications in tiny engineering field, such as micro-fabrication, microfluidics and so on.^96^

In the field of environmental protection, light-driven microrobots can effectively treat sewage and clean surfaces. Because it does not require the involvement of chemical fuels, it is more practical, more eco-friendly and more economical.^97^

In the biomedical field, light-driven microrobots are expected to be used in surgery, targeted drug delivery and single-cell manipulation. We believe that the introduction of microrobots is more direct and efficient, which can reach the lesion, and reduce the damage caused by the invasive treatment of traditional rigid medical devices.^98^

However, there are still some key challenges for light-driven microrobots, which hinder their development and application. For light-driven microrobots rely on accurate optical micromanipulation technology, whose driving force is in the range of nano-newtons to pico-newtons, so it is not enough to drive larger and heavier microrobots. Moreover, the equipment of most optical micromanipulation techniques are complex and expensive, preventing light-driven microrobots from being widely used.^99^

Last but not least, the spatial adjustment ability of light in space is limited. Magnetic field and sound field can be used to control the three-dimensional spatial position of the object, while the light field mainly controls object in the two-dimensional space.^100^ Additionally, light can drive objects to move smoothly in a transparent medium, but restricted in an opaque medium, such as blood. It makes the application of light-driven microrobots in medical treatment challenges. However, we still believe that under the joint efforts of researchers from all over the world, and the integration of various driving mechanisms, the field of light-driven microrobot will continue to flourish and its applications will continue to expand.

## Author contributions

Yibao Chen completed the topic selection of the paper, Yingnan Gao completed the literature reading and writing, Xiaowen Wang completed the final revision of the paper.

## Conflicts of interest

There are no conflicts to declare.

## Supplementary Material
